# Integrated analysis of bulk and single-cell RNA sequencing reveals the interaction of PKP1 and tumor-infiltrating B cells and their therapeutic potential for nasopharyngeal carcinoma

**DOI:** 10.3389/fgene.2022.935749

**Published:** 2022-09-14

**Authors:** Yu-Mei Huang, Lin-Qian Wang, Ying Liu, Fa-Qing Tang, Wen-Ling Zhang

**Affiliations:** ^1^ Department of Clinical Laboratory, The Third Xiangya Hospital, Central South University, Changsha, Hunan, China; ^2^ Clinical Laboratory of Hunan Cancer Hospital, The Affiliated Cancer Hospital of Xiangya School of Medicine, Central South University, Hunan Key Laboratory of Oncotarget Gene, Changsha, Hunan, China

**Keywords:** PKP1, tumor-infiltrating lymphocyte-B cells, bulk RNA sequencing, single-cell RNA sequencing, nasopharyngeal carcinoma

## Abstract

Immunotherapy is an individualized therapeutic strategy for nasopharyngeal carcinoma (NPC). However, few molecular targets are clinically satisfactory. This work aimed to integrate bulk and single-cell RNA sequencing data to identify novel biomarkers involved in NPC. We performed differentially expressed gene (DEG) analysis, Gene Ontology (GO) enrichment, Kyoto Encyclopedia of Genes and Genomes (KEGG) pathway analysis, and immune cell infiltration analysis prior to correlation analysis of the identified genes and immune cells and further assessed the prognostic effects of the biomarkers and immune cells in NPC. As a result, PKP1, a potential molecular biomarker associated with immune infiltration, and tumor-infiltrating lymphocyte-B cells (TIL-Bs) were identified as promising therapeutic targets for NPC. Importantly, immunohistochemistry (IHC) validated that PKP1 protein expression was mainly found in NPC cells rather than noncancerous cells. In addition, the tumor microenvironment (TME) of NPC was characterized by the infiltration of more dendritic cells (DCs) and γδT cells but fewer B cells. Our results suggest that the interaction of PKP1 and TIL-B cells is involved in NPC development. It is possible that TIL-B cells produce immunoglobulin G (IgG) to tumor antigens, such as PKP1, or viral antigens, including EBV and HPV, to execute antitumor ability through DC and T cells. In response, NPC cells express proteins such as PKP1 (absent in normal nasopharynx) to induce myeloid-derived suppressor cell (MDSC) expansion, which subsequently impairs the proliferation of B cells and results in B-cell death by generating iNOS and NOX2. In summary, our findings provide a potential therapeutic strategy for NPC by disrupting the interaction of PKP1 and TIL-Bs in the TME.

## Introduction

Nasopharyngeal carcinoma (NPC) is a unique head and neck malignancy that originates from the nasopharynx. NPC exhibits a distinct geographic distribution in which >70% of new cases originate in southern China and southeast Asia (Chen et al., 2019; Wong et al., 2021). Since the location of NPC is anatomically hidden and patients often exhibit no precursors or specific clinical symptoms, the discovery of the disease is usually delayed until it has reached an advanced stage, accompanied by cervical lymph nodes with or without distant metastasis (Bossi et al., 2021; Siak et al., 2021; Xie et al., 2022). Therefore, there is an urgent need for a highly sensitive and specific molecular biomarker for the early diagnosis of disease, prognosis, and prediction of recurrence and metastasis. Despite the efforts of scientists and doctors toward this goal through technologies such as genomics, transcriptomics, proteomics and metabolomics (Zhang et al., 2021; Siak et al., 2021), no such satisfactory biomarkers are currently available.

Gene expression profiling of tumor tissue is an important resource for simulating tumor microenvironment (TME) conditions and identifying potential biomarkers. However, NPC tissues are difficult to obtain. Sometimes, the primary tumor is not visible at endoscopy (King et al., 2019). Fortunately, with the high throughput and affordable price of sequencing technology, a large amount of sequencing data based on various kinds of samples have been generated. Most of these data are shared by researchers on datasets with free access such as Gene Expression Omnibus (GEO). One of the advantages of using these public data is that we can combine homogeneous sample data from different sources. In this way, the sample size is enlarged, and correspondingly, the reliability of the conclusion is enhanced. Here, we utilized bulk RNA sequencing (bulk RNA-seq) data from GEO and single-cell RNA sequencing (scRNA-seq) results from a web portal to investigate the molecular biomarkers and TME of NPC and to explore the underlying mechanism.

## Materials and methods

The design of this study is shown by a flow chart (Supplementary Figure S1).

### Information on nasopharyngeal carcinoma sequencing data from Gene Expression Omnibus

We retrieved the expression profiles of NPC samples produced by high-throughput sequencing from the GEO database (http://www.ncbi.nlm.nih.gov/geo/). Then, three datasets (GSE68799, GSE118719 and GSE102349) were obtained (Table 1). Because GSE68799 and GSE118719 included both tumor and nontumor tissues, we downloaded their raw clean data to probe the differentially expressed genes (DEGs) and the hub genes. We also downloaded the normalized (CPM and TMM) data of GSE102349 submitted to GEO by researchers. Considering that GSE102349 introduced relatively complete clinical information, including disease progression, we used it for survival and diagnostic analysis.

**TABLE 1:** The basic information of the three datasets.

Datasets name	GSE68799	GSE118719	GSE102349
Platform	GPL11154	GPL20301	GPL11154
Sequencer	Illumina HiSeq 2000	Illumina HiSeq 4000	Illumina HiSeq 2000
NPC	42	7	113
non-NPC	4	4	0

NPC, Nasopharyngeal carcinoma.

### RNA-sequencing raw data analysis

RNA sequencing raw data were downloaded by sratoolkit. Quality control was carried out by fastaqc software, a quality control tool for high-throughput sequencing data (https://www.bioinformatics.babraham.ac.uk/projects/fastqc/). The raw sequence reads were aligned to the human genome GRCh38.103 by the star aligner (star-2.7.8a) (Dobin et al., 2012). Then, the gene expression matrix of read counts was quantified by merging from ReadsPerGene text generated by star.

This work was carried out and relied on computing resources from the High-Performance Computing Center of Central South University, China (CentOS Linux release 7.5.1804).

### Screening of the common differentially expressed genes

The raw read count matrix from the previous step was normalized and used to perform differential gene analysis by the R package DESeq2 (https://bioconductor.org/packages/release/bioc/html/DESeq2.html). Principal component analysis (PCA) was implemented to detect the batch effect. Genes with an adjusted *p* value (FDR) < 0.05 and | log2FC | > 2 were considered significant. Volcano maps of all significant genes were plotted by the easy-to-use data visualization web server ImageGP (Chen et al., 2022). The online web tool Venny 2.1 was employed to overlap the upregulated and downregulated DEGs of the two datasets.

### Gene annotation and enrichment analysis of the differentially expressed genes and identification of the hub genes

Gene Ontology (GO) functional and Kyoto Encyclopedia of Genes and Genomes (KEGG) pathway analyses of the common DEGs were carried out by the R package clusterProfiler. FDR <0.05 was considered to indicate a significant difference. The GO analysis consisted of biological process (BP), cellular component (CC) and molecular function (MF).

Metascape integrates more than 40 bioinformatics databases that allow biologists to easily analyze comprehensive data through a simple one-click interface. It provides enrichment analysis of biological pathways, structure analysis of protein‒protein interaction (PPI) networks and abundant gene annotation functions (Zhou et al., 2019). The PPI network was constructed by metascape, which utilizes physical protein‒protein interactions from BioGrid as the main data source, integrating interactome datasets, including InWeb_IM and OmniPath. In addition, an MCODE identification algorithm was adopted by Metascape to automatically extract the protein complexes and obtain the core genes.

### Prognostic significance analysis

The GSE102349 dataset was exploited for prognostic significance analysis in this study due to the availability of survival information. The survminer package was used to identify the recent survival analysis threshold. Then, the progression-free survival (PFS) of NPC patients was evaluated via the R package survival by the two-sided log-rank test, and *p* values *<* 0.05 were considered statistically significant.

### Expression and location analysis

It was unclear what types of cells possessed the genes that could predict progression of NPC, so we applied a website portal (db.cngb.org/npcatlas) based on Chen and his colleagues’ study on NPC where researchers can analyze and visualize the single-cell data easily and for free (Chen et al., 2020). We input the gene names to identify whether they were located in epithelial cells or immune cells.

Furthermore, immunohistochemistry (IHC) was performed to validate the expression and location of PKP1 protein in ten primary NPC specimens. The study was approved by the Medical Ethics Committee of Xiangya Hospital Central South University (No: 202207389). Briefly, 4-μm formalin-fixed, paraffin-embedded (FFPE) NPC tissue sections were deparaffinized in xylene and then rehydrated in sequentially increasing dilutions of alcohol. Antigen retrieval was conducted in EGTA (pH 9.0, AR-0541, Dingguo Changsheng, Beijing, China) at 95°C using a water bath. After that, the sections were blocked with 5% BSA at 37°C for 30 min followed by incubation overnight at 4°C with rabbit anti-PKP1 antibody (1:250, ab154622, Abcam, Cambridge, MA, United States). Next, the sections were blocked using an endogenous peroxidase blocker and subsequently incubated with a mouse/rabbit enhanced polymer detection system (PV-9000, ZSGB-BIO, Beijing, China) as described in the instruction manual. After that, the sections were treated with chromogen (DAB) for 5 min at room temperature. The sections were dried and prepared for optical microscope observation.

### Immune cell infiltration analysis

The outstanding platform of Immune Cell Abundance Identifier (ImmuCellAI, http://bioinfo.life.hust.edu.cn/ImmuCellAI#!/) was utilized to predict the abundance of 18 T-cell subtypes and 6 other immune cells, B cells, NK cells, dendritic cells (DCs), macrophage cells, monocyte cells and neutrophil cells, with the given gene expression data, including bulk RNA-Seq and microarray data (Miao et al., 2020). We merged the GSE68799 and GSE118719 datasets and assayed their batch effects. Then, ImmuCellAI was implemented to estimate the proportion of immune cells infiltrated in NPC tissues from this merged dataset as well as the GSE102349 dataset. Then, we compared the differences in immune cell infiltration between the NPC and nontumor samples from the merged data by the Kruskal‒Wallis test. Meanwhile, box plots were introduced to visualize the data distribution. A *p* value < 0.05 was considered statistically significant. Survival analysis of different cell types was also performed on the GSE102349 dataset. A *p* value < 0.1 was considered statistically significant.

### Genes and immune cells correlation analysis

The R package ggcorrplot was adopted to disclose the relationships between the expression levels of the selected genes and the immune marker sets that constituted the first layer of the immune cells defined by ImmuCellAI, including DC cells, B cells, monocyte cells, macrophage cells, NK cells, neutrophil cells, CD4^+^ T cells, CD8^+^ T cells, NKT cells, and gamma delta + T cells (γδT). *p* values were determined by Spearman’s rank correlation test with a statistical significance of *p <* 0.05.

## Results

### Identification of differentially expressed genes in nasopharyngeal carcinoma

**FIGURE 1:**
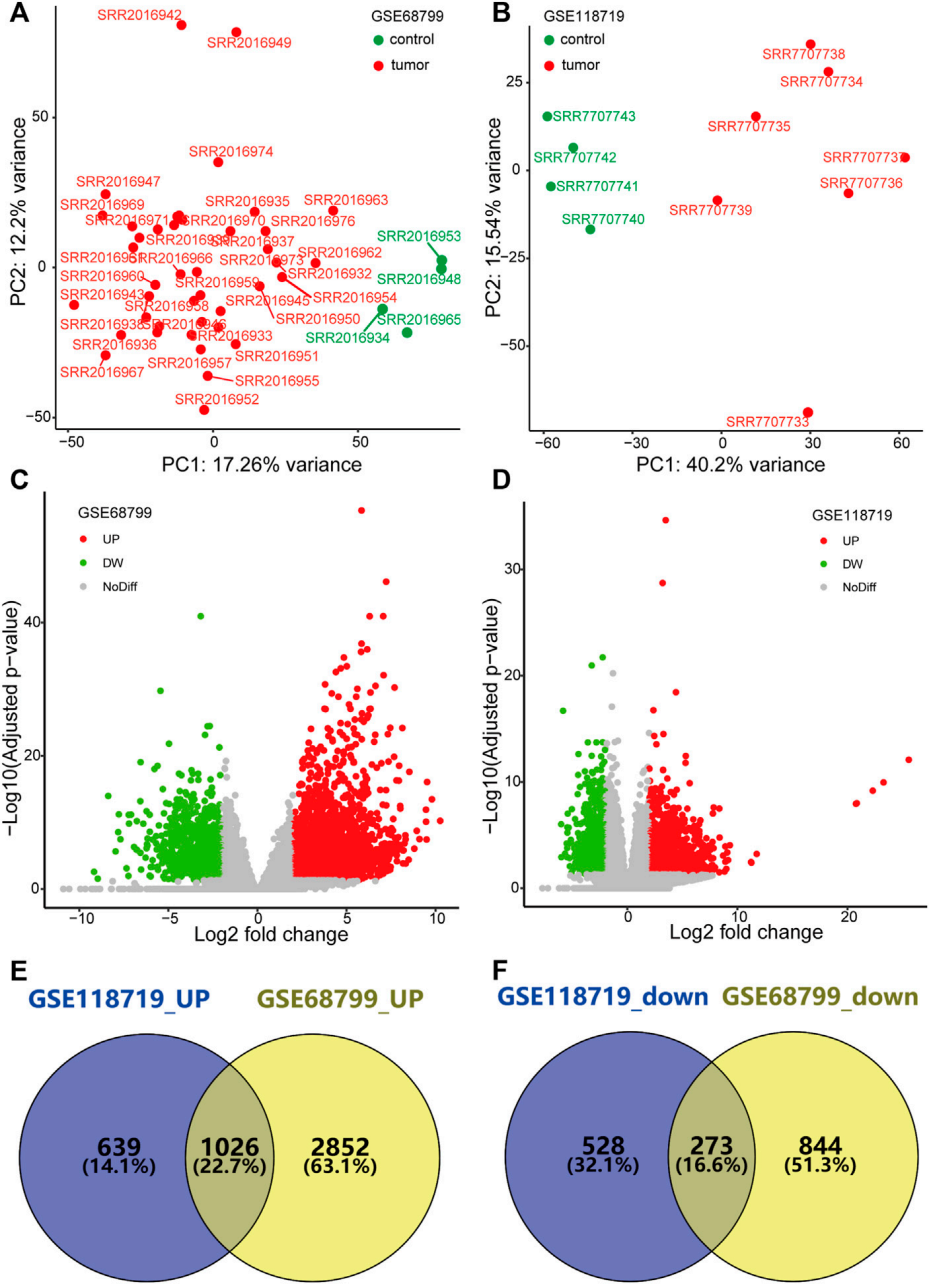
Identification of differentially expressed genes (DEGs). **(A,B)** PCA plots show that genes from GSE68799 and GSE118719 were mainly classified into the tumor cluster and the control cluster. **(C,D)** Volcano plots present the distribution of DEGs with thresholds of FDR<0.05 and |log2 FC| > 2 in GEO datasets GSE68799 and GSE118719. **(E,F)** Venn diagrams show the common genes of upregulated and downregulated DEGs from the two datasets.

PCA showed that data from GSE68799 and GSE118719, after normalization, were mainly classified into the tumor cluster and the control cluster, indicating that they were appropriate for further analysis (Figures 1A,B). With the criteria of FDR*<*0.05 and | log2 FC | *>* 2, DEGs in the GSE68799 dataset were identified, which consisted of 3,878 upregulated and 1,117 downregulated genes, as displayed by a volcano plot in Figure 1C. In the GSE118719 dataset, 1,665 genes were found to be upregulated and 801genes were downregulated (Figure 1D). Then, we overlapped the upregulated and downregulated DEGs from the two datasets by the Venn tool and obtained 1026 upregulated and 273 downregulated genes (Figures 1E,F).

### Enrichment analysis of the differentially expressed genes and identification of the hub genes

**FIGURE 2:**
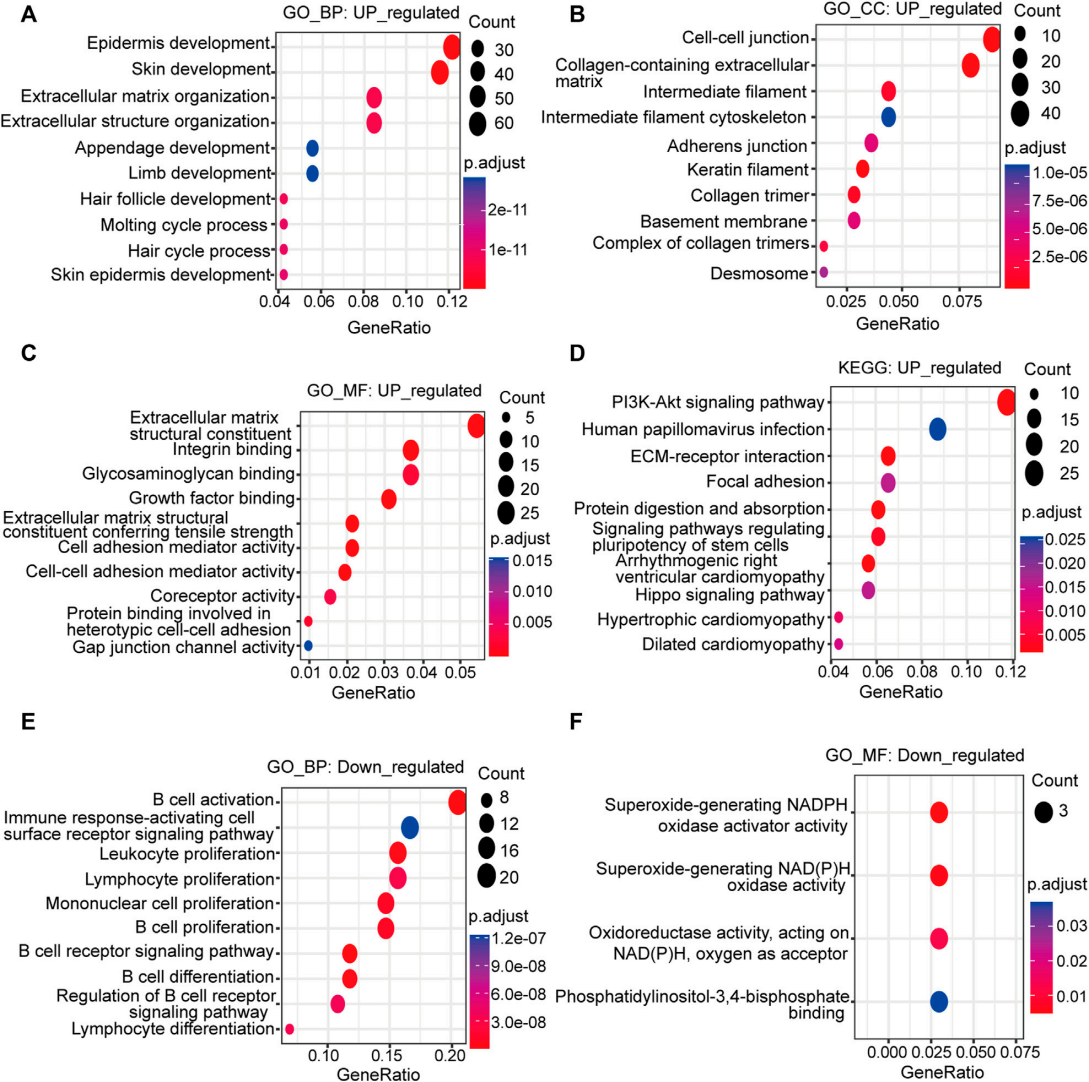
Functional analysis of the upregulated and downregulated DEGs. Bubble plots depict the GO and KEGG signaling pathway enrichment analysis. The size of the bubble represents the gene count, and the color of the dot represents the adjusted *p* value. Upregulated: **(A)** BP; **(B)** CC; **(C)** MF; **(D)** KEGG pathway analysis; downregulated: **(E)** BP; **(F)** MF; GO: Gene Ontology; KEGG: Kyoto Encyclopedia of Genes and Genomes; BP: biological process; CC: cellular component; MF: molecular function.

GO analysis revealed that the upregulated DEGs relevant to BP were preferentially enriched in skin development, epidermis development, extracellular matrix organization, extracellular structure organization and hair follicle development (Figure 2A). The upregulated DEGs involved in CC were markedly enriched in cell‒cell junctions, collagen-containing extracellular matrix, keratin filaments, collagen trimers and intermediate filaments (Figure 2B). The upregulated DEGs associated with MF were predominantly clustered in extracellular matrix structural constituents, extracellular matrix structural constituents conferring tensile strength, integrin binding, cell adhesion mediator activity and cell‒cell adhesion mediator activity (Figure 2C). KEGG analysis revealed that the PI3K-Akt signaling pathway, human papillomavirus infection, MAPK signaling pathway, calcium signaling pathway, and ECM-receptor interaction were the primary enriched signaling pathways (Figure 2D).

The downregulated DEGs related to BP were enriched in B-cell activation, B-cell proliferation, the B-cell receptor signaling pathway, leukocyte proliferation, lymphocyte proliferation and mononuclear cell proliferation (Figure 2E). It could be inferred from the BP of the downregulated DEGs that they were mainly concentrated in the activation and proliferation of immune cells, especially B cells. The main MF involved was NADPH oxidase activity (Figure 2F). The CC was the external side of the plasma membrane. The KEGG pathway was the B-cell receptor signaling pathway.

### Construction of a protein‒protein interaction network and module analysis

**FIGURE 3:**
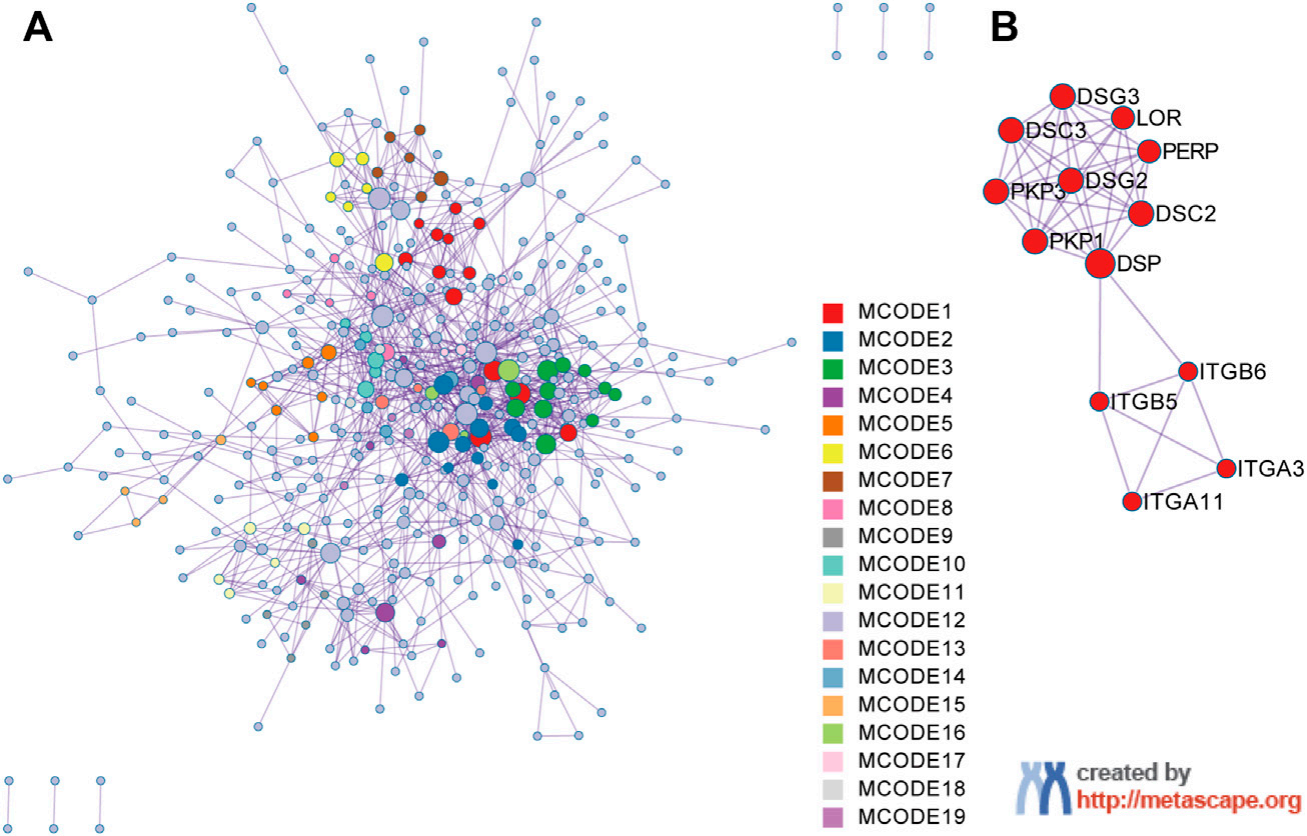
Protein‒protein interaction (PPI) network construction and MCODE component identification by Metascape. **(A)** PPI network constructed with the 1026 upregulated DEGs. **(B)** The first module identified from the PPI network using the molecular complex detection (MCODE) method with a score of 3.31. Each color represents a unique MCODE module.

The PPI network of the upregulated DEGs was constructed by the Metascape web server (Figure 3A; Supplementary Table S1). The significant modules of the DEGs were also automatically extracted by using the molecular complex detection (MCODE) identification algorithm. We then chose the first-presented module that contained most of the genes (13 genes) and 223 edges for further analysis (Figure 3B).

### Identification of prognostic markers for nasopharyngeal carcinoma

We used the calculated cut point for PFS analysis (cut points are shown in Supplementary Table S2).

**FIGURE 4:**
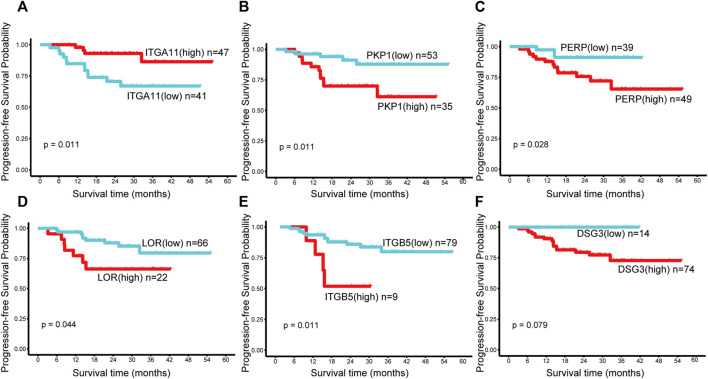
Kaplan–Meier survival curves of the genes in the first module in NPC. Progression-free survival (PFS) by high and low **(A)** *ITGA11*, **(B)** *PKP1*, **(C)** *PERP*, **(D)** *LOR*, **(E)** *ITGB5*, **(F)** *DSG3* mRNA expression. A *p* value < 0.05 was considered statistically significant. The best cut-off values which were determined by the R package Survminer were selected to divide the cases into high and low expression groups.

The high expression of *ITGA11* indicated good prognosis in NPC patients, while the high expression of *PKP1*, *PERP*, *LOR* and *ITGB5* showed poor PFS (Figures 4A–E). Nevertheless, there was no significant correlation between *DSG3*, *ITGA3*, *PKP3*, *DSG2*, *DSC2*, *DSC3*, *DSP*, and *ITGB6* expression and survival (Figure 4F, only shows *DSG3* as representative).

### Gene location analysis

**FIGURE 5:**
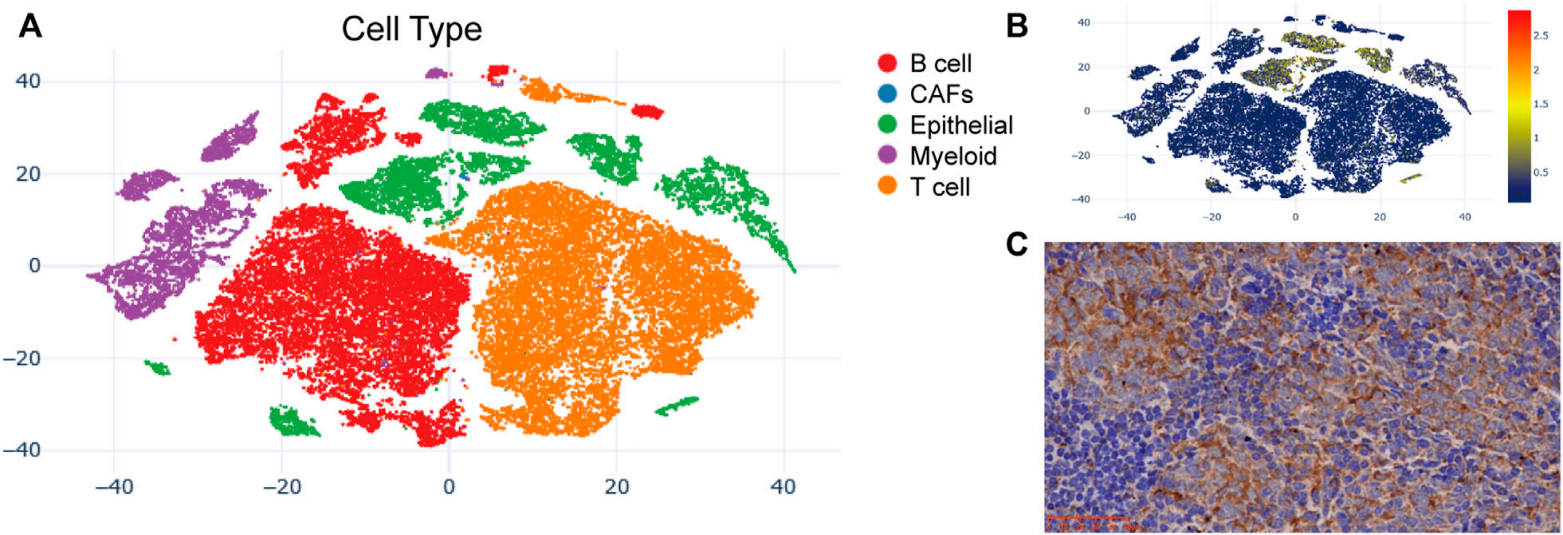
PKP1 expression analysis in NPC patients. **(A)** The t-SNE plots of cells with each cell color-coded to indicate the different cell types; **(B)** The mRNA expression of *PKP1* in cells defined above; **(C)** Immunohistochemical staining of PKP1 protein was performed in 10 NPC tissues. A representative image is shown. Scale bars are shown in red.

The t-SNE cell plot displayed five cell types with each cell color coded, including B cells, cancer associated fibroblasts (CAFs), epithelial cells, myeloid cells, and T cells (Figure 5A). *PKP1* was predominantly found in epithelial cells (Figure 5B). *PERP* was mainly distributed in epithelial cells at a relatively high level. In contrast, the expression of *ITGA11*, *LOR*, and *ITGB5* was very low in each cell type. Then, we further confirmed the protein expression of PKP1. As a result, IHC validated the distribution and expression of PKP1 protein in NPC tissues. Weak to strong cytoplasmic positivity was observed mainly in NPC cells rather than in noncancerous cells (Figure 5C).

### Immune cell infiltration and prognostic significance analysis

**FIGURE 6:**
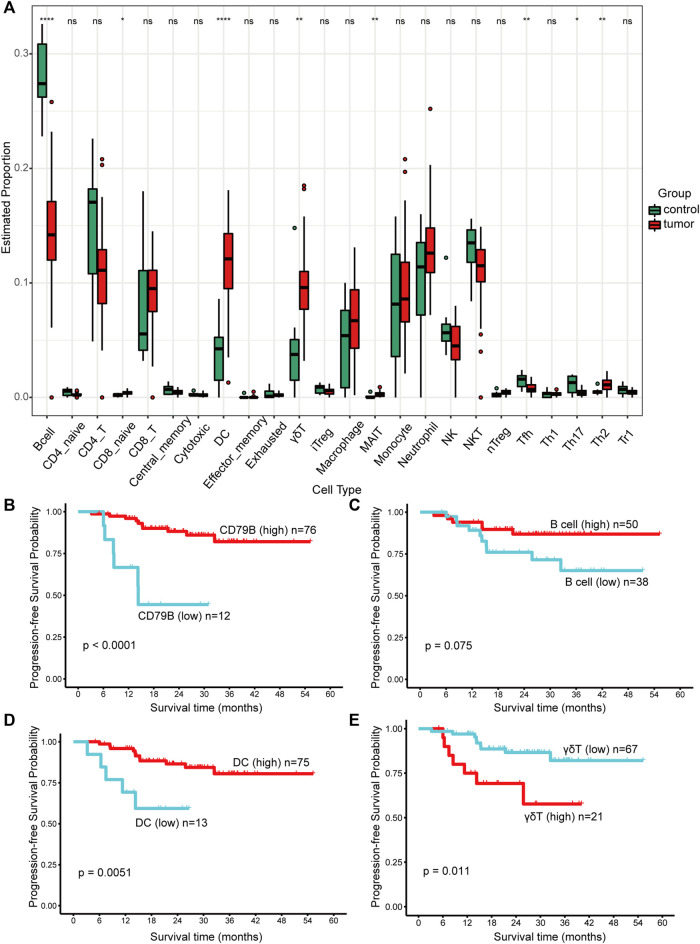
The proportions of immune cells and their prognostic significance analysis. **(A)** The proportions of immune cells infiltrated in control and tumor tissues. Data were analyzed using the Kruskal‒Wallis test. A *p* value > 0.05 was considered not statistically significant and represented as ns, while a *p* value ≤ 0.05 was represented as *, ≤0.01 as **, ≤0.001 as ***, and ≤0.0001 as ****. **(B–E)** Kaplan–Meier survival analysis of progression-free survival (PFS). The best cut-off values which were determined by the R package Survminer were selected to divide the cases into high and low expression groups. A *p* value < 0.1 was considered statistically significant.

Among the 24 cell types, B cells, Tfh cells, and Th17 cells achieved higher proportions in nontumor tissues than in NPC tissues. More naïve CD8^+^ cells, DCs, γδT cells, MAIT cells, and Th2 cells infiltrated NPC (Figure 6A). Cells infiltrated in tissues were mainly in layer 1, including DCs, B cells, monocytes, macrophages, NK cells, neutrophils, CD4^+^ T cells, CD8^+^ T cells, NKT cells, and γδT cells (Figure 6A). With respect to the extremely low proportion of the cells in layer 2 (maximum 2.4%, maximum median 1.025%), analysis was not conducted to avoid statistical errors.

Significantly, the infiltration condition of the B cells was consistent with the DEG results, in which CD79B was downregulated in NPC. Further analysis revealed that both high expression of CD79B (*p* < 0.0001, Figure 6B) and B cells (*p* = 0.075, Figure 6C) could predict favorable prognosis in NPC. Of note, a longer progression time was observed in patients with higher DC infiltration (*p* = 0.0051, Figure 6D). Patients with higher γδT cell infiltration were more likely to progress (*p* = 0.011, Figure 6E). However, monocytes, macrophages, NK cells, neutrophils, CD4^+^ T cells, CD8^+^ T cells, and NKT cells were not significantly associated with PFS (figures not shown).

### Correlations between the genes and immune cells

**FIGURE 7:**
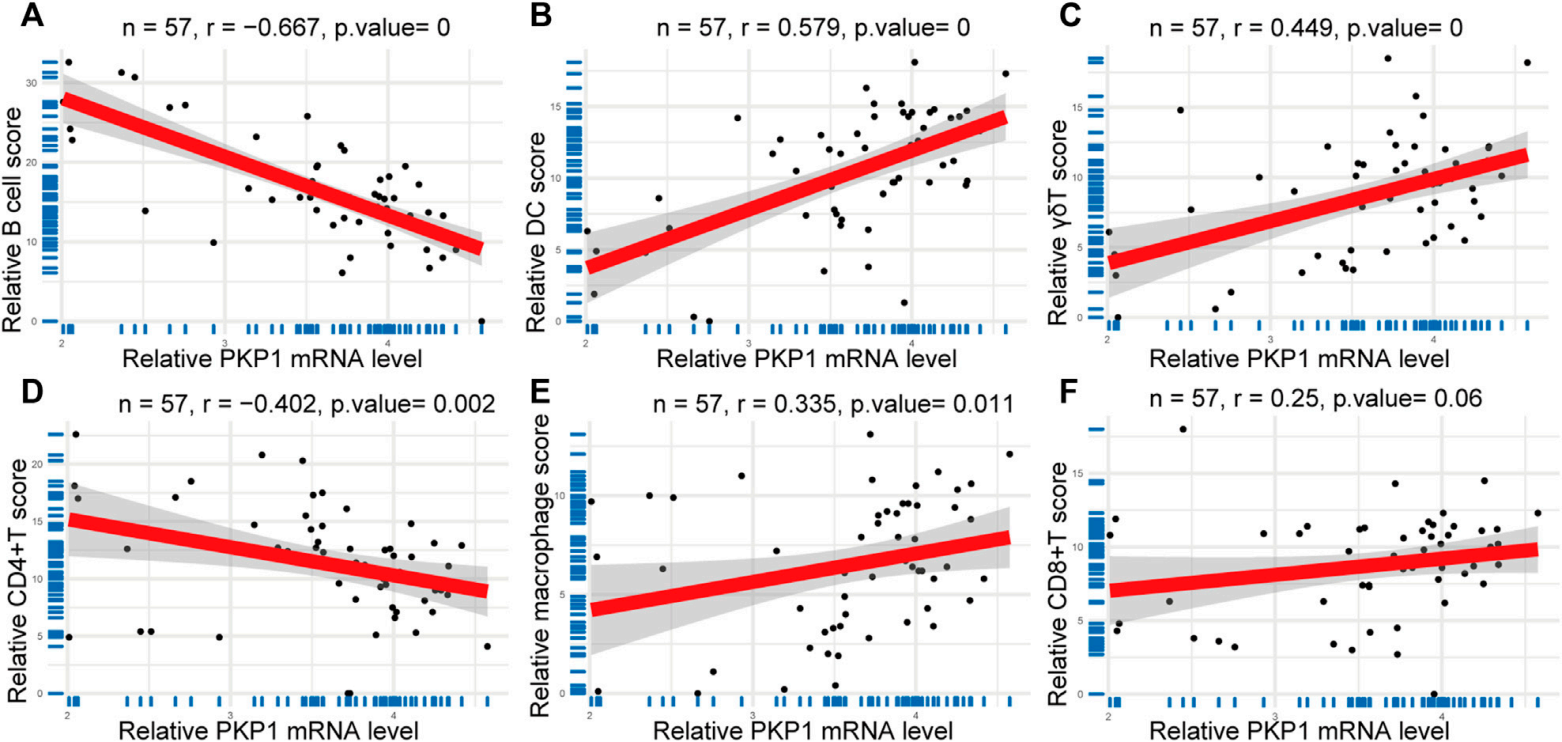
Correlations between *PKP1* and immune cells. **(A–E)** The expression of relative *PKP1* mRNA was significantly correlated with B cells, DCs, γδTs, CD4^+^ T cells, and macrophages. **(F)** A representative that the expression of relative *PKP1* mRNA had no significant correlation with CD8^+^ T cells, monocytes, NK cells, neutrophils, and NKT cells. *p* values were calculated by Spearman’s rank correlation test with a statistical significance of *p* < 0.05.

The expression of relative *PKP1* mRNA was significantly correlated with B cells (r = −0.667, *p* = 0), DCs (r = 0.579, *p* = 0), macrophages (r = 0.335, *p* = 0.011), γδTs (r = 0.449, *p* = 0), and CD4^+^ T cells (r = −0.402, *p* = 0.002) (Figures 7A–E). However, there was no significant correlation between *PKP1* and CD8^+^ T cells, monocytes, NK cells, neutrophils, and NKT cells (Figure 7F as a representative).

## Discussion

High-throughput bulk RNA-Seq and scRNA-Seq are revolutionary approaches to cancer research that contribute to revealing the heterogeneity between tumors (Levy and Boone, 2019; Zhang et al., 2021). scRNA-Seq analyzes a single-cell suspension, which is, on the downside, an obstacle for this technology due to the difficulty of obtaining a single-cell sample and the loss of certain cell types (Mints and Tirosh, 2020). Whereas bulk RNA-seq, just as its name implies, deals with a population of cells in bulk samples, it is incapable of uncovering the diversity of the cells within a tumor like scRNA-Seq. The sensitivity of this technology is greatly improved as the yield of cells increases, together with a boost in data generation. To make full use of these data, several excellent algorithms have been developed to estimate the proportions of the cell types in a mixed cell population based on gene expression datasets, such as bulk RNA-Seq and microarray data. Among them, ImmuCellAI, CIBERSORT, and TIMER are outstanding representatives (Newman et al., 2019; Li et al., 2020; Miao et al., 2020). NPC is characterized by massive lymphocyte infiltration, which is significantly related to the progression of the cancer and the effect of immunotherapy (Chen et al., 2020). However, the mechanism of immune cell infiltration and the genes capable of assessing prognosis and immune cell infiltration in NPC remain unknown. Our study intended to integrate bulk RNA-seq data with scRNA-Seq information to identify biomarkers and assess immune cell infiltration in NPC.

Our GO and KEGG pathway analysis results demonstrated that the upregulated DEGs were preferentially present in skin and epidermal development, which is consistent with their epithelial characteristics (Chen et al., 2019). Moreover, the results of GO and KEGG analyses of downregulated DEGs and the proportions of infiltrating immune cells also revealed that B cells were significantly less infiltrated in the NPC microenvironment than in normal tissues. The above results reflected that the tumor tissues used in this study acquired from the two gene sets GSE68799 and GSE118719, on the one hand, were of good quality, suggesting that the results obtained from these datasets would be reliable; on the other hand, the reduced infiltration of B cells was indicative of the features of the TME in NPC. Combined with the immune cell infiltration analysis, we initially depicted the TME landscape of NPC tissues, which were infiltrated with more DC and γδT cells but fewer B cells in the first layer, while in the second layer, there were more CD8 naïve cells, MAITs, and Th2 cells but fewer Tfh and Th17 cells. Furthermore, survival analysis indicated that B-cell and DC infiltration were protective factors for NPC patients, whereas infiltration of γδT cells was a risk factor.

To date, cancer immunotherapy has focused on the T-cell compartment. Although, apart from T cells, other immune subsets have not been widely studied, several reports have concluded that B cells in the TME contribute to antitumor responses (Cabrita et al., 2020; Helmink et al., 2020; Petitprez et al., 2020; Dyugay et al., 2022). In NPC patients, B cells in tertiary lymphoid structures (TLSs) could be recruited to improve survival (Li et al., 2021). In addition, NPC has a strong etiological association with EBV infection (Baloche et al., 2020; Jin et al., 2020; Tsang et al., 2020). Since B cells are the major host of EBVs, which can transmit through oral secretions and colonize epithelial cells in the oropharynx, the virus shuttles between B cells and epithelial cells (Houen and Trier, 2020). This finding reminds us that B cells are indeed important in the development of NPC. Intratumoral B cells can produce immunoglobulin G (IgG) antibodies to enhance the internalization of tumor antigens presented by DCs, and subsequently activate tumor-reactive T cells. This mechanism is believed to have potent antitumor immunity, with evidence that tumors in mice have been successfully eradicated (Carmi et al., 2015). In addition, human papillomavirus (HPV) expression has been reported to be linked to NPC (Huang et al., 2018), which is supported by our KEGG results. There is evidence of the infiltration of HPV-specific B cells in the TME of head and neck cancer, which can secrete HPV-specific IgG antibodies, contributing to antitumor immunity through indirect effects such as enhancing antigen cross-presentation or the generation and maintenance of HPV-specific T-cell responses (Wieland et al., 2021). In light of our TME landscape of NPC, we boldly speculate that tumor-infiltrating B cells in NPC produce specific antibodies and then exert antitumor effects through DCs and T cells.

Correspondingly, tumor cells struggle to resist attacks from the immune system in a variety of ways, such as the overexpression of some molecules as weapons. For example, latent membrane protein 1 (LMP1), the EBV principal oncoprotein and essential for cell malignant transformation and immortalization, induces myeloid-derived suppressor cell (MDSC) expansion to suppress immune responses (Kieser and Sterz, 2015; Cai et al., 2017). MDSCs are the major cause of tumor immunosuppression, so they come naturally to us. Many studies have proven that MDSCs are propagated in the TME of various tumors, including NPC (Li et al., 2015; Pyzer et al., 2016; Zhang et al., 2019; Yang et al., 2020). Moreover, MDSCs can restrain the proliferation of B cells and induce B-cell death by expressing iNOS and NADPH oxidase (NOX2), which are relevant to the production of nitric oxide (NO) and reactive oxygen species (ROS), respectively (Gabrilovich and Nagaraj, 2009; Raffaghello and Bianchi, 2014; Lelis et al., 2017). Furthermore, Wang et al. confirmed that MDSCs could suppress B-cell proliferation and impair B-cell responses in lung cancer (Wang et al., 2018). Interestingly, our study found that the activity of NADPH oxidase was the predominantly enriched MF of the downregulated genes and that B cells were the markedly reduced cell type. Therefore, it is reasonable for us to surmise that NPC cells can recruit MDSCs to kill B cells through the expression of molecules such as LMP or the potential biomarker PKP1, which was determined in our analysis and subsequently discussed.

PKP1 is an important accessory desmosomal plaque protein that participates in desmoglein (DSG) membranous availability (Fuchs et al., 2019). It is involved in cell growth, motility, migration, and apoptosis (Weiske et al., 2001; Sobolik-Delmaire et al., 2007; Wolf et al., 2010). PKP1 has been shown to be downregulated in lung cancer and predict favorable clinical outcomes (Haase et al., 2019). While PKP1 is rarely expressed in the nasopharynx, as reported by integrated proteomics in GeneCards: Protein expression in normal tissues and cell lines from ProteomicsDB, MaxQB, and MOPED. Indeed, our study found that it was upregulated and was predominantly present in NPC cells but not in noncancerous cells. Our analysis further revealed that the mRNA levels of *PKP1* predicted poor prognosis of NPC and were positively correlated with the infiltration levels of DCs, γδTs, and macrophages and negatively correlated with B cells and CD4^+^ T cells in NPC.

**FIGURE 8:**
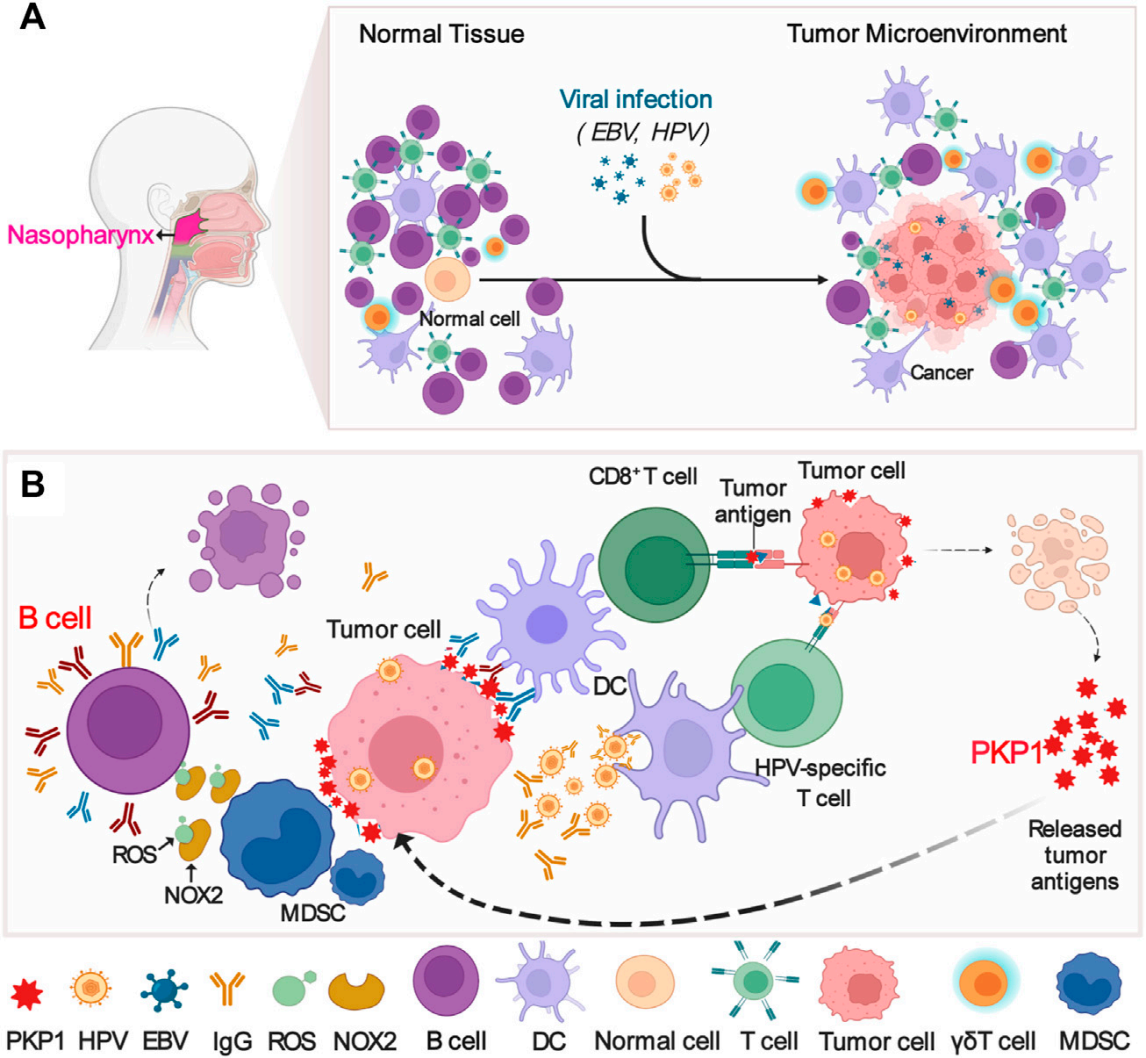
Possible mechanism diagram of the interaction between PKP1 and TIL-Bs in NPC microenvironments. **(A)** EBV and HPV infection results in NPC. **(B)** The interaction between PKP1 and TIL-Bs. TIL-Bs produce IgG to kill tumors, and NPC cells fight back. TIL-Bs: tumor-infiltrating lymphocyte-B cells. This diagram is only a proposal of the mechanism that warrants further validation. Created with BioRender.com.

Overall, we depicted a possible mechanism diagram as follows (Figure 8): EBV and/or HPV infect nasopharyngeal epithelial cells and then lead to NPC for undefined reasons (Figure 8A). Tumor-infiltrating lymphocyte-B cells (TIL-Bs) produce IgG to tumor antigens such as PKP1 to assist dendritic cell internalization of tumor antigens, which subsequently activates T cells in response to tumor cells; alternatively, HPV-specific IgG may be generated to enhance antigen cross-presentation or provoke and maintain the HPV-specific T-cell reaction. In response, NPC cells struggle to resist attacks from the immune system. NPC cells express proteins such as PKP1 (absent in the normal nasopharynx) to induce MDSCs expansion, which subsequently impairs the proliferation of B cells and results in B-cell death by expressing iNOS and NOX2 (Figure 8B). In summary, our results provide a potential therapeutic strategy for NPC by disrupting the interaction of PKP1 and TIL-B cells in the TME and a theoretical basis for better understanding the mechanism of NPC development.

## Conclusion

In summary, this work indicates that *PKP1* can be a candidate gene for assessing the immune infiltration levels in NPC and a potential therapeutic target for NPC. More importantly, inducing TIL-Bs in the TME might be an effective anti-NPC approach. This paper also provides a theoretical basis as well as a new perspective to help elucidate the underlying mechanism of NPC development and progression.

## Data Availability

The datasets presented in this study can be found in online repositories. The names of the repository/repositories and accession number(s) can be found in the article/Supplementary Material.
