# Supplementary figures and images for: Integrated analysis of bulk and single-cell RNA sequencing reveals the interaction of PKP1 and tumor-infiltrating B cells and their therapeutic potential for nasopharyngeal carcinoma
Source: Front Genet. 2022 Sep 14;13:935749. doi: 10.3389/fgene.2022.935749 (PMC9515358; doi:10.3389/fgene.2022.935749)

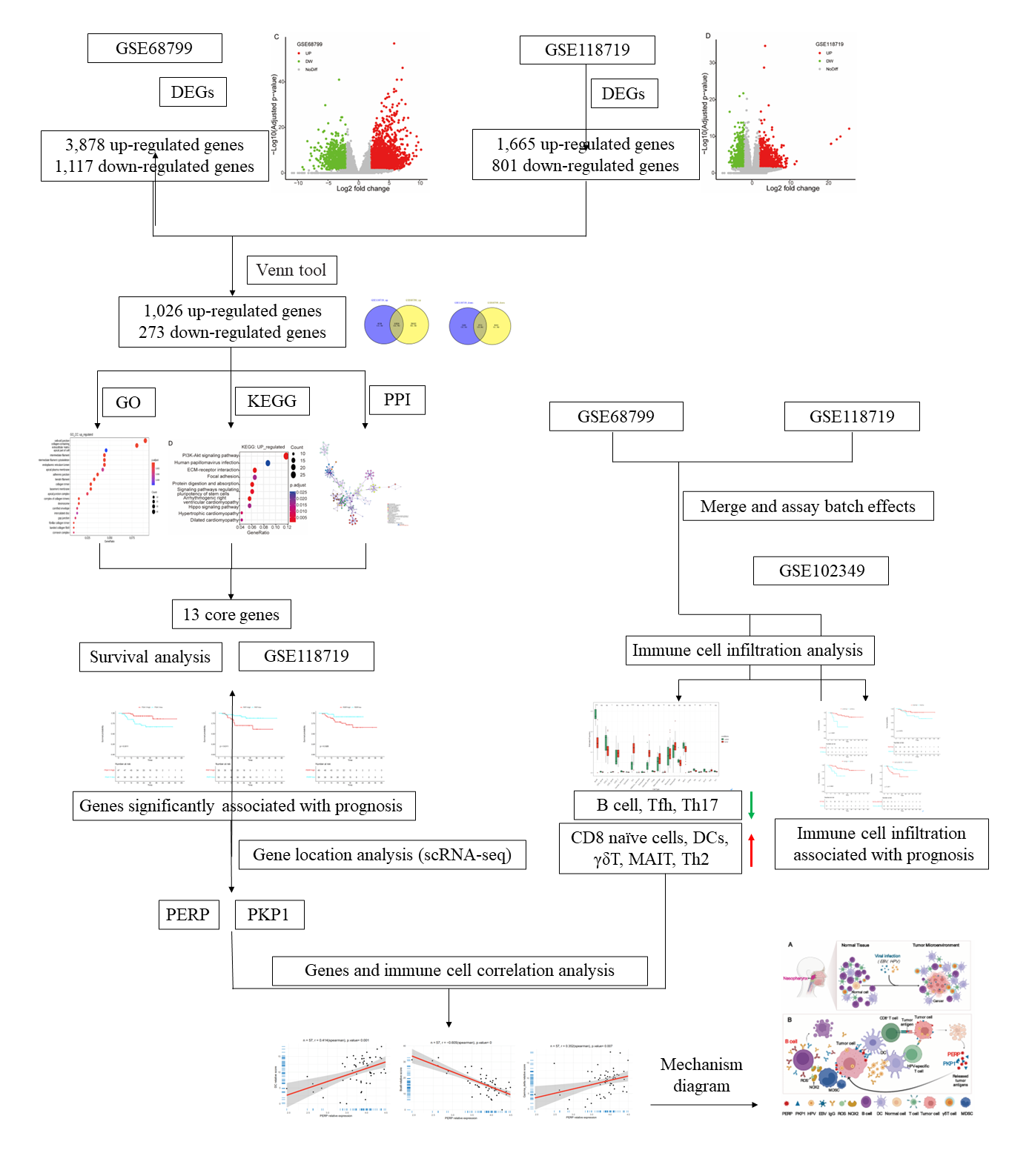

Supplement: Supplementary file 1 [file Image1.tif]
